# 

**Published:** 1880-12

**Authors:** 


### CONTENTS:

ORIGINAL COMMUNICATIONS:

I. Genital Irritation, together with some Remarks on the Hygiene of the Genital Organs in Young Children. By Roswell Park, M.D. ..... 562

II. Ovarian Cysts complicated with Hernia, Hæmorrhoids, Prolapsus Vaginæ and Fibroid Tumors of the Uterus—Successful Ovariotomy. By T P. Seeley, M.D. ..... 570

III. Natural Selection in Diseases. By H. D. Valin, M.D. ..... 579

IV. Caries of the Superior Maxilla. By Truman W. Brophy, M.D. ..... 582

V. Abstract of an Address delivered before the Alumni Association of Rush Medical College. By Prof. DeLaskie Miller ..... 586

CLINICAL REPORTS:

*Notes from Private Practice:*

VI. Cleft of the Hard and Soft Palate Closed by Operation, with Produciion of Bone in the Palatine Vault ..... 590

DOMESTIC CORRESPONDENCE:

VII. New York Letter ..... 593

SOCIETY REPORTS:

VIII. Aurora Medical Society ..... 600

REVIEWS AND BOOK NOTICES ..... 602

SELECTIONS:

On Perityphlitis ..... 605

Some Practical Remarks on Chronic Rheumatism ..... 619

Treatment of Sprains ..... 644

On Wasting or "Simple Atrophy" as it Occurs in Young Children from Insufficient Nourishment ..... 648

The Treatment of Asthma ..... 656

Aneurism of the Arch of the Aorta, Cured by Rest, Restricted Diet and Iodide of Potassium and Ergot ..... 663

OBITUARY:

F. H. Davis, M.D. ..... 667

Calvin G. Goodrich, M.D. ..... 669

ITEMS ..... 589, 592, 599, 665, 670

ANNOUNCEMENTS FOR THE MONTH ..... 672

Notice to Correspondents ..... xxi advts.

